# Effects of recreational soccer in men with prostate cancer undergoing androgen deprivation therapy: study protocol for the ‘FC Prostate’ randomized controlled trial

**DOI:** 10.1186/1471-2407-13-595

**Published:** 2013-12-13

**Authors:** Jacob Uth, Jakob Friis Schmidt, Jesper Frank Christensen, Therese Hornstrup, Lars Juel Andersen, Peter Riis Hansen, Karl Bang Christensen, Lars Louis Andersen, Eva Wulff Helge, Klaus Brasso, Mikael Rørth, Peter Krustrup, Julie Midtgaard

**Affiliations:** 1 The University Hospitals Centre for Health Care Research (UCSF), Copenhagen University Hospital Rigshospitalet, Blegdamsvej 9, Copenhagen 2100, Denmark; 2 Department of Nutrition, Exercise and Sports, University of Copenhagen, Nørre Allé 51, Copenhagen 2200, Denmark; 3 Sport and Health Sciences, College of Life and Environmental Sciences, University of Exeter, Prince of Wales Road, Exeter, Devon, UK; 4 Department of Cardiology, Copenhagen University Hospital Gentofte Hospital, Niels Andersens Vej 65, Hellerup 2900, Denmark; 5 Department of Urology and Copenhagen Prostate Cancer Center, Copenhagen University Hospital Rigshospitalet, Blegdamsvej 9, Copenhagen 2100, Denmark; 6 The National Research Centre for the Working Environment, Lersø Parkallé 105, Copenhagen 2100, Denmark; 7 Department of Biostatistics, University of Copenhagen, Øster Farimagsgade 5, Copenhagen 1014, Denmark; 8 Department of Oncology, Copenhagen University Hospital Rigshospitalet, Blegdamsvej 9, 2100 Copenhagen, Denmark; 9 Department of Cardiology, Herlev University Hospital, Herlev Ringvej 75, Herlev 2730, Denmark; 10 Department of Clinical Medicine, Faculty of Health and Medical Sciences, University of Copenhagen, Blegdamsvej 3B, Copenhagen 2200, Denmark

**Keywords:** Prostate cancer, Androgen deprivation therapy, Physical exercise, Soccer training, Rehabilitation, Body composition, Cardiovascular function

## Abstract

**Background:**

Androgen deprivation therapy (ADT) is a cornerstone in the treatment of advanced prostate cancer. Adverse musculoskeletal and cardiovascular effects of ADT are widely reported and investigations into the potential of exercise to ameliorate the effects of treatment are warranted. The ‘Football Club (FC) Prostate’ study is a randomized trial comparing the effects of soccer training with standard treatment approaches on body composition, cardiovascular function, physical function parameters, glucose tolerance, bone health, and patient-reported outcomes in men undergoing ADT for prostate cancer.

**Methods/Design:**

Using a single-center randomized controlled design, 80 men with histologically confirmed locally advanced or disseminated prostate cancer undergoing ADT for 6 months or more at The Copenhagen University Hospital will be enrolled on this trial. After baseline assessments eligible participants will be randomly assigned to a soccer training group or a control group receiving usual care. The soccer intervention will consist of 12 weeks of training 2–3 times/week for 45–60 min after which the assessment protocol will be repeated. Soccer training will then continue bi-weekly for an additional 20 weeks at the end of which all measures will be repeated to allow for additional analyses of long-term effects. The primary endpoint is changes in lean body mass from baseline to 12 weeks assessed by dual X-ray absorptiometry scan. Secondary endpoints include changes of cardiovascular, metabolic, and physical function parameters, as well as markers of bone metabolism and patient-reported outcomes.

**Discussion:**

The FC Prostate trial will assess the safety and efficacy of a novel soccer-training approach to cancer rehabilitation on a number of clinically important health outcomes in men with advanced prostate cancer during ADT. The results may pave the way for innovative, community-based interventions in the approach to treating prostate cancer.

**Trial registration:**

ClinicalTrials.gov: NCT01711892

## Background

Prostate Cancer (PCa) is the most common non-cutaneous malignancy in men, with 650,000 estimated new cases per year in the developed world [1]. Androgen deprivation therapy (ADT) remains a cornerstone of PCa management, with approximately 50% of men diagnosed with PCa undergoing ADT at some point in time [2]. ADT is administered with curative intent before and 2–3 years after radiotherapy for locally advanced disease [3], or as continuous palliative treatment for disseminated disease [4]. The 15 year relative survival rate now exceeds 90% for all PCa stages combined and there has been a steady increase in the number of PCa survivors [5], partly attributable to the greater anti-neoplastic efficacy of ADT and radiotherapy in combination.

While ADT contributes to improved life expectancy, it is also associated with significant adverse effects, including loss of lean body mass (LBM), decreased bone mineral density (BMD), poor functional performance, increased fat percentage, insulin resistance, and increased risk of fractures [6-10]. The combination of ADT-induced adverse effects and subsequent changes in health behavior, i.e., physical inactivity and deconditioning, may predispose PCa patients to serious morbidity, including elevated risk of cardiovascular and metabolic disorders, leading to increased mortality [11,12]. Therefore, interventions aimed at counteracting ADT-induced adverse effects may result in profound survival benefits for patients with PCa.

Physical exercise is emerging as a promising supplementary treatment strategy in the oncology setting, with capacity to improve aerobic fitness, muscle strength, body composition, quality of life (QoL) and physical function, and to reduce fatigue [13,14]*.* Indeed, such improvements have been reported after physical exercise in studies of PCa patients undergoing ADT. Galvão et al. found that 12 weeks of combined resistance and aerobic training improved muscle mass, muscular strength, physical function and balance [15]. In agreement with these results, Segal et al. [16] found that a 24 week program of aerobic exercise combined with resistance training mitigated fatigue and maintained aerobic fitness in PCa patients undergoing radiotherapy with or without concurrent ADT. Although data from these and other randomized controlled trials (RCTs) [17-19] suggest that physical exercise interventions have considerable potential in counteracting treatment-related side-effects, important questions remain unanswered. First, the duration of interventions to date has been relatively brief (i.e., 12 or 24 weeks), and consequently little is known about whether effects of training can be maintained or even improved in the longer term. Secondly, the effects of exercise on numerous physiologic outcomes, i.e., bone metabolism, glucose tolerance, cardiac structure and function and peripheral vascular function, have yet to be described in PCa patients. Thirdly, information about the safety, feasibility and efficacy of exercise interventions for PCa patients with advanced stage disease involving bone metastases is scarce, as only one previous study has included this population [19]. Finally it is not known whether results demonstrated in previous exercise studies can be reproduced in alternative and non-clinical settings, e.g., organized team sports [14].

Therefore the purpose of the present study is to investigate 1) the effects of 12 weeks of recreational soccer on body composition, fitness, cardiac structure and function, peripheral vascular function, blood pressure, physical function parameters, postural balance, muscle strength, glucose tolerance, insulin sensitivity, and markers of inflammation and bone metabolism and 2) whether potential physiological and patient-reported effects of the 12 week soccer training intervention can be maintained or improved further with an additional 20 weeks training at a reduced training volume. The primary study endpoint is changes in LBM from baseline to 12 weeks.

## Methods/Design

### Study design

This study is a two-armed RCT, with one group playing soccer (intervention group) and a waiting-list control group, who is offered participation in the intervention after the 8 months study period. The study has been approved by the Danish National Committee on Biomedical Research Ethics for the Capital Region (registration number H-3-2011-131) and written informed consent will be obtained from all participants before any study procedures are undertaken.

### Blinding and masking of data

Blinding of patients and soccer instructors in this kind of study is not possible. All data will be entered into a secure web server immediately after collection and will not be available to study personnel at subsequent tests. At the termination of the study a statistician blinded to treatment assignment will perform all analyses before disclosing any study outcome data to the study coordinator and researchers involved in the study.

### Study population

We aim to include and randomize 80 men with histologically confirmed advanced or locally advanced PCa presenting at Copenhagen Prostate Cancer Center and Dept. of Urology, Copenhagen University Hospital Rigshospitalet, Denmark. Patients aged < 76 years who have received ADT for at least 6 months will be invited to attend meetings which will outline the purpose of the study, and offer more detailed information about the investigations involved and the soccer intervention.

Assessments will be performed at the following locations: The Panum Institute Copenhagen (dual-energy X-ray absorptiometry [DXA] scans), The August Krogh Building at the Department of Nutrition, Exercise and Sports (cardio respiratory fitness test, peripheral vascular function tests, electrocardiogram), The National Research Centre for the Working Environment (balance, jump, chair stand and stair climbing tests) and Department of Cardiology, Copenhagen University Hospital, Gentofte Hospital, Denmark (echocardiography). All training sessions will take place at The Department of Nutrition, Exercise and Sports, University of Copenhagen.

### Inclusion criteria

•Patients with locally advanced or advanced PCa managed with medical or surgical ADT for at least 6 months.

•Age between 18 and 76 years.

•Ability to read and understand Danish.

•Signed informed consent.

### Exclusion criteria

•WHO performance level > 1.

•Osteoporosis (T-score < −2.5).

•Activity limiting pain from bone metastasis.

•Cardiovascular or pulmonary disorders (e.g., arrhythmias, ischemic heart disease, unregulated high blood pressure, chronic obstructive lung disease).

•Anticoagulant therapy.

•Abnormal screening blood samples (hemoglobin <7.0 mM, creatinine >150 mikroM, thrombocytes <150,000/mikroL).

•Abnormal liver function.

•Coagulopathy.

•Malignant disease other than PCa.

•Current or scheduled chemotherapy.

### Randomization

**Figure 1:**
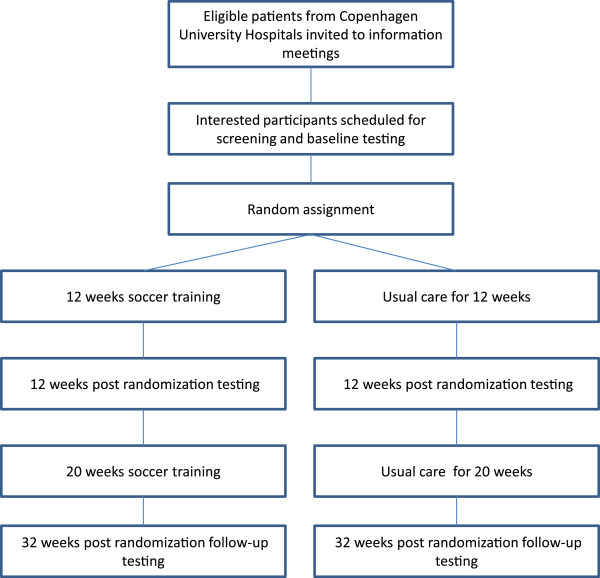
CONSORT diagram.

After successful completion of all baseline assessments participants are randomized 1:1 to the soccer intervention or control group. The randomization process will be conducted by a research consultant at The Copenhagen Trial Unit who has no other involvement in the study. The study flowchart is presented in Figure 1.

### Treatment arms

#### Intervention group

**Figure 2:**
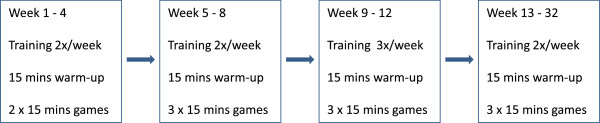
Duration and frequency of soccer training sessions during the study period.

Participants in the intervention group will practice soccer for 12 weeks two-three times weekly. An experienced soccer instructor will be in charge of all training sessions. During weeks 1–4 training will consist of two weekly sessions of 15 min of warm-up exercises (running, dribbling, passing, shooting, balance and muscle strength exercises) followed by 2 × 15 min of 5–7 a-side games. In weeks 5–8 the duration of each session will increase to 3 × 15-min games after the warm-up, and in weeks 9–12 there will be three weekly training sessions of the same duration. After 12 weeks all assessments will be repeated. Participants in the intervention group will then continue bi-weekly supervised training for an additional 20 weeks at the end of which all assessments will be repeated to allow for additional analysis of long-term effects (Figure 2). Training will take place on a natural grass pitch. In adverse weather conditions (i.e., < 5°C or heavy rain) training will be performed indoors. Participants will be told to avoid hard tackles and other actions that carry a risk of injury.

#### Control group

Participants in the control group will be encouraged to maintain their baseline physical activity level. However, for ethical reasons, this advice will not be enforced, since increasing physical activity levels in general is considered beneficial to health.

### Study assessments

All assessments will take place at baseline, and after 12 weeks and 32 weeks. Measurement of body composition, peripheral vascular function, glucose tolerance, blood pressure, and blood markers will be performed in the morning after an overnight fast. Subjects will be instructed to avoid intake of medication, caffeine and vitamins, and to abstain from tobacco use for 12 h prior to the above mentioned tests and to avoid strenuous physical activities 48 h prior to all examinations.

#### Primary study endpoint

The primary study endpoint is the change in LBM as determined by whole body DXA-scan (iDXA, Lunar Corporation, Madison, WI, USA) according to standard procedures.

#### Secondary study endpoints

Secondary outcomes include body composition, measures of physical functioning, assessment of cardiovascular and metabolic function, blood test values and patient-reported outcomes.

### Body composition

Assessment of total body BMC and areal BMD as well as android, gynoid and total body fat mass will be derived from the whole body DXA scan. Visceral fat mass will be evaluated using the iDXA CoreScan software (Lunar Corporation, Madison, WI, USA). BMC and areal BMD of the hips and lumbar spine will be derived from separate DXA scans. Height will be measured by a stadiometer, body weight will be measured with a digital platform scale and body mass index will be calculated (weight in kg/(height in m)^2^).

### Waist- and hip circumference

Waist circumference will be measured around the abdomen at the level of the belly button, and the hip circumference will be measured at the widest part of the hips and hip to waist ratio will be calculated [20].

### Physical function tests

#### Maximal oxygen uptake

Two hours after consuming a normal breakfast participants will conduct a submaximal walking test on a treadmill and an incremental test to exhaustion on a cycle ergometer. The submaximal test will consist of 4 min of walking on a treadmill at 4.5 km/h to determine oxygen uptake, respiratory exchange ratio and heart rate during an activity similar to that of daily living. After 4 min of passive rest, the incremental cycle test will start with 4 min cycling at 40 W, with a self-chosen cadence in the range of 70–90 rpm, after which the load will increase by 20 W each min until volitional exhaustion. Oxygen uptake, respiratory exchange ratio (RER) and ventilation will be determined by pulmonary gas exchange measurements (MasterScreen CPX, Viasys Healthcare, St Paul, Minnesota, USA). The physiological criteria for approval of the maximal oxygen uptake (VO_2_max) test will be RER ≥ 1.05 and leveling off on the VO_2_ curve with an increase of <1 ml O_2_/min/kg with an increase in work load of 20 W [21]. Heart rate will be determined in 5 s intervals throughout the incremental test by a Polar Team System chest belt (Polar Oy, Kempele, Finland). VO_2_max and maximal heart rate (HRmax) will be defined as the highest oxygen uptake and heart rate values obtained over a 30 and 15 s period, respectively.

#### Flamingo balance test

Postural balance will be assessed with a modified single-leg flamingo balance test [22]. Subjects are instructed to stand on one foot on a 3 cm wide and 5 cm high metal bar with their eyes open for one min. Subjects are permitted to move their arms and non-standing leg to assist balancing. The number of falls will be counted and used as a measure of postural balance.

#### Assessment of postural sway

Subjects will be asked to stand on a force platform (AMTI R6-1000, Watertown, MA, USA), arms crossed over the chest, and instructed to look at a 10 cm^2^ circle placed 2.5 meters away from the platform at a height of 1.65 m. Vertical ground reaction force (Fz), anterior-posterior moment (Mx) and medio-lateral moment (My) will be sampled using custom made Matlab (Mathworks) acquisition software at 100 Hz (16 bit A/D conversion, DT9804, Data translation, Marlboro, MA, USA). The Fz-, Mx- and My-signals will be digitally low-pass filtered with a 4^th^ order zero-lag Butterworth filter (8 Hz cutoff) [23]. Displacement of the center of pressure will be calculated as (x,y) = (x_0_ + My/Fz, y_0_ + Mx/Fz), where (x_0,_y_0_) is the geometrical center of the plate. Balance will be tested in three positions: 1) bilateral (60 s): feet close together with skin contact both at heels and bases of hallux; 2) unilateral (15 s): base of hallux of the free foot placed on medial malleolus of the standing leg; 3) tandem stand (15 s): both feet on the force plate with base of hallux of one foot placed next to medial malleolus of the other foot. Bilateral standing is performed once, unilateral and tandem standing are performed in triplicate and the trials with the smallest sway area will be used for further analysis. The data acquisition method has been previously described in detail elsewhere [24].

#### Counter movement jump (CMJ)

On a force platform (AMTI R6-1000, Watertown, MA, USA) subjects will perform standard CMJs with hands placed on the hips. On the signal “go” the subject is instructed to bend their knees and jump as high as they can without moving their hands. The vertical force signal (Fz) obtained during the jump will be used to calculate the offset impulse, i.e., the area under the force-time curve, which will then be converted to velocity by dividing by body mass, and finally converted to jump height based on the relationship between kinetic and potential energy [25]. Subjects will perform three jumps separated by 30-s resting periods and the highest jump height (cm above ground) will be used in subsequent analysis.

#### Sit-to-stand test

Using a chair fixed to the ground with a seat 45 cm above the ground subjects will be instructed to sit in the middle of the chair, back straight, arms crossed over their chest, feet flat on the floor. A mechanical contact in the seat is connected to a computer which automatically counts the number of rises. Correct standing technique will be demonstrated first slowly, then quickly. Subjects will be allowed to practice for two-three repetitions before the start of the test. On the signal “go” the subject will be asked to stand, then return to the seated position, as many times as possible in 30 s [26].

#### Stair climbing

Subjects will be instructed to climb up one flight of a staircase (9 steps, 0.175 m each) as fast as they safely can, taking one stair at a time, without holding the handrails [27]. The time taken by the subject between touching the first step to reaching the last step will be measured manually with a stopwatch.

#### Muscle strength

Dynamic concentric muscle strength for the knee extensors will be assessed with the one repetition maximum (1RM) test measured in 2.5 kg intervals. After a standardized warm-up the test load will start at 15 kg and resistance will gradually be increased until failure. The rest period between each attempt is 30 seconds. The maximum weight lifted through a full range of motion will be recorded as 1 RM [28].

### Cardiovascular and metabolic function

#### Echocardiography

Comprehensive transthoracic echocardiography will be performed on a GE Vivid 9 ultrasound machine with a 2.5 MHz transducer (GE Healthcare, Horton, Norway). The examination will be performed with the subjects resting in lateral supine position in a dark room by two experienced echocardiographers blinded for group allocation. All examinations will be analyzed off-line in random order, using the Echo Pac software version BT 11.0 by an independent and blinded echocardiographer. The full echocardiographic protocol has been described elsewhere [29]. Cardiac structure will be evaluated from parasternal long axis 2-D recordings at the mid-ventricular level with measurement of left ventricular (LV) end-diastolic diameter (LVEDD), interventricular septal wall thickness (IVST) and posterior wall thickness (PWT). LV mass is calculated from the formula 0.832 [1.05 [(LVID + IVST + PWT)^3^] − (LVID)^3^] and indexed according to body surface area and LV volumes. LV ejection fraction will be evaluated with Simpson’s biplane method.

Right ventricular function will be evaluated as tricuspid annular plane systolic excursion. Diastolic function will be measured as peak transmitral inflow velocity in early (E) and late (A) diastole and the corresponding E/A-ratio and pulsed analyses of tissue Doppler Imaging (TDI) of diastolic velocities E´ and A´ will be obtained with a 5-mm pulsed (TDI) sample volume placed in the lateral, septal, anterior and inferior plane of the mitral annulus in the 2- and 4-chamber apical views. TDI peak systolic velocity (S´; cm/s) will also be measured. The values of E´ will be reported as an average of the septal and lateral early peak diastolic velocities and E/E´ will be calculated as a measure of left ventricular filling pressure. Two-D color tissue Doppler will be evaluated from six basal segments of septal, lateral, anterior, inferior, posterior, and anterior septal walls of the apical 2- and 4-chamber and long axis and values will be averaged. Measurements will include S´, E´ and A´. Diastolic dysfunction will be graded as previously described [29]. LV longitudinal systolic function will be evaluated by 2D- speckle tracking analysis and longitudinal 2-D global strain will be estimated using automated functional imaging. LV longitudinal systolic shortening (LV displacement) will be evaluated using tissue tracking as described previously by others [30].

#### Peripheral vascular function

Measurements of the reactive hyperemic index (RHI) and the augmentation index, respectively, will be measured with peripheral arterial tonometry (PAT) under standardized conditions in a quiet dark room. A pneumatic probe will be placed on the tip of each index finger and connected to a plethysmographic device (EndoPat-2000, Itamar Medical Ltd, Caesarea, Israel). After this PAT measurements will be made before and during reactive hyperemia as previously described [31] in order to derive RHI, a measure of microvascular endothelial function, and the augmentation index, a measure of arterial stiffness, normalized to a heart rate of 75 bpm, respectively.

#### Oral glucose tolerance test (OGTT)

The participants will be asked to drink 0.5 L of a 15% glucose solution within a 5-min period. Blood samples will be collected prior to the 75 g glucose intake as well as after 15, 30, 60, and 120 min to measure plasma glucose and insulin. Glucose tolerance will be measured by the 2 hour value and the area under the curve for glucose. To determine whole body insulin sensitivity the insulin sensitivity index (ISI) proposed by Matsuda and Defronzo will be calculated from fasting and mean plasma glucose and insulin concentrations obtained from the measuring time points during the OGTT [32].

#### Blood pressure

After a 1½ hr resting period during the OGTT, blood pressure will be measured with a digital sphygmomanometer (OMRON-M7) 5 times on the left arm at 2 min intervals. The average of the 5 measurements will be recorded for subsequent analysis.

#### Blood sampling and analyses

Blood samples will be obtained from a cubital vein and serve as a screening tool at baseline. Thus, hemoglobin and iron status will be measured to avoid inclusion of patients with anemia (Sysmex XE-2100, Sysmex America, Inc., Lincolnshire, IL, USA). Coagulation markers of International Normalized Ratio, activated partial thromboplastin time (APTT) and trombocytes will be measured to rule out coagulopathy (ILS ACL TOP, Instrumentation Laboratory, 1930 Zaventem, Belgium and Sysmex XE-2100, Sysmex America, Inc., Lincolnshire, IL, USA). Serum concentrations of creatinine will be measured to rule out kidney disorders and levels of aminotransferases, alkaline phosphatase and bilirubine to rule out liver disorders (MODULAR analyzers, Roche Diagnostics, Mannheim, Germany). In addition all blood samples will be analyzed at baseline, 12 and 32 weeks for total cholesterol, low-density lipoprotein cholesterol, high-density lipoprotein cholesterol, triglycerides, and glycosylated hemoglobin, respectively, by automated analyzers (Cobas Fara, Roche, Neuilly sur Seine, France) using enzymatic kits (Roche Diagnostics, Mannheim, Germany, and Tosoh G7, Tosoh Europe, Tessenderlo, Belgium). All of the above mentioned blood markers will be analyzed in the Department of Clinical Biochemistry at Copenhagen University Hospital Rigshospitalet, Denmark.

All samples will also be analyzed for bone markers, including procollagen type I C propeptide, osteocalcin, C-terminal telopeptide, tartrate-resistant acid phosphatase 5b and leptin, using ELISA and AlphaLISA apparatus (PerkinElmer, Cambridge, United Kingdom) at the Scientific Laboratory at the University of Exeter, United Kingdom.

### Patient-reported outcomes

Information on socio-demographic and lifestyle characteristics will be collected at baseline [33]. Health-related quality of life outcomes will be measured using the eight sub scales of the Medical Outcomes Study Short Form [34] and the 15 subscales of the European Organization for Research and Treatment of Cancer (EORTC QLQ-C30) [35]. Diagnosis-specific symptoms and side-effects will be measured with the supplement EORTC QLQ-PR25. Anxiety and Depression will be measured with the two subscales of the Hospital Anxiety and Depression Scale [36,37]. Social support and network will be measured with The Multidimensional Scale of Perceived Social Support [38]. Leisure time physical activity level will be examined using a self-administrated questionnaire classifying patients in the following groups: I) sedentary; II) walking or cycling for pleasure; III) regular physical exercise at least 3 hrs per week; or IV) intense physical activity more than 4 hours per week. The Physical Activity Scale will be used to assess average weekly physical activity of sleep, work, and leisure time [39].

### Medical history and status

Detailed information about the time of PCa diagnosis, disease stage, PSA level at the time of diagnosis, duration of ADT at baseline, previous radiation treatment or surgery and pre-existing comorbidities will be obtained from the participants’ medical records.

### Tracking and monitoring during soccer training

#### Heart rate

Participants will wear heart rate monitors (Polar Electro Oy, Kempele, Finland) to determine heart rate zones and intensity.

#### Activity profile

GPS monitors (GPSport, Melbourne, Australia) will be worn by participants in week 2 and 11 to record standing, walking and running times, as well as running speeds and distance covered.

#### Perceived exertion

In week 2 and 11 of the intervention participants’ perceived exertion and experience of flow will be determined with Visual Analogue Scales [40].

#### Adherence

Attendance and reasons for non-attendance of training sessions (e.g., muscle soreness, injury, in- or outpatient visits to the hospital) will be recorded in a training log book.

#### Adverse events

Serious adverse events occurring during the training will be reported immediately to the Unit for Patient Safety in the Capital Region of Denmark. Minor events related to physical contact or stumbling can occur during training, and only pain and soreness persisting for more than 24 h will be recorded.

### Statistical considerations

#### Sample size calculations

Since recreational soccer has not previously been applied as a rehabilitation strategy for men with PCa, the possible effect size on LBM is unknown. However, Krustrup et al. [41] have shown that the effect of soccer training on LBM is comparable to that of progressive resistance exercise [42]. In men with PCa undergoing ADT progressive resistance exercise has yielded increases in LBM of 0.7 kg after 12 weeks of training [15]. To detect a 0.7 kg difference in LBM between the groups, assuming a standard deviation (SD) of 1.0 kg, 34 patients are needed in each group with a significance level (two-sided) of 5% and a power of 80%. Due to possible dropouts we plan to include 40 patients in each group.

#### Data analyses

Data entry will be undertaken using a secure web server and statistical analysis will be performed using Statistical Analysis Systems (SAS) version 9.2. The statistician will prepare results with no knowledge of the randomization coding. The primary endpoint will be reported as a two-sample t-test comparing change scores in the two randomization groups. Significance level will be set at 0.05.

Regarding secondary outcomes, the continuous variables, i.e., VO_2_max, HRmax, waist and hip circumferences, CMJ, and stair climbing parametres, and the patient reported outcomes, respectively, will be reported as either means with corresponding 95% confidence limits or as medians and interquartile range (IQR). For count data, i.e., the Flamingo balance test and the sit-to-stand test, Poisson regression will be used, and categorical data, i.e., single questionnaire items, will be reported as proportions and compared across randomization groups using chi-squared tests or logistic regression.

## Discussion

Adverse treatment side-effects of ADT for PCa patients include loss of LBM, increased fat percentage and increased risk of myocardial infarction [11], fractures [43] and diabetes [10], as well as reduced QoL [44]. Interventions aimed at mitigating these side effects are both warranted and important for patient well-being [14].

The current study will provide a comprehensive investigation into the effects of a relatively brief (12 weeks) and medium-term (32 weeks) exposure to physical exercise on numerous physiological outcomes including body composition, cardiovascular function, bone health, insulin sensitivity, mobility, muscle strength, balance and patient reported outcomes such as QoL in PCa patients undergoing ADT. Recreational soccer will be used as a unique and novel rehabilitation initiative and, to the best of our knowledge, this is the first time soccer has been proposed as a complementary intervention in the treatment of cancer. This is also the first study to examine cardiac function in PCa patients with comprehensive echocardiography, and to monitor the impact of exercise training in PCa patients on cardiac function and peripheral vascular function. Animal studies have provided evidence that exercise training may counteract left ventricular dysfunction associated with ADT in rodents [45]. Our study is therefore likely to provide important and novel information about cardiovascular health in PCa patients undergoing ADT.

The choice of soccer as an intervention is based on a number of considerations. Firstly, participation in sport is increasingly recognized as important for public health [46] and recent evidence from a large prospective cohort study shows that participation in organized sport is associated with reduced mortality (hazard ratio = 0.71; 95% CI = 0.56, 0.91) [47]. Secondly, soccer is considered the most prominent team sport in the world, with more than 270 million active sports club players [48] and most Danish men have played the game. Thirdly, recent studies have shown that recreational soccer induces beneficial musculoskeletal, metabolic and cardiovascular adaptations in healthy untrained young men [49], middle-aged men with hypertension [50,51], premenopausal women [52] and middle-aged men with type 2 diabetes [29]. The positive effects obtained after 12–14 weeks of soccer training in the studies with healthy untrained young men and premenopausal women were maintained with a reduced training volume beyond a one-year period following the intervention [53,54]. Mean heart rates of 80-85% of HRmax and numerous (>190/h) high intensity actions, i.e. dribbles, shots, turns, jumps, sprints, accelerations, decelerations and tackles, may explain why soccer effectively stimulates both aerobic and anaerobic energy delivery systems [55,56]. In relation to bone health, a topic of particular concern in the PCa population, studies have found that 12–14 weeks of soccer training significantly increases lower extremity bone mass [49] and volumetric BMD in the tibia [52] and results in marked increases in plasma levels of osteocalcin [57]. Intense and diverse movements resulting in the generation of large ground reaction forces are hypothesized to account for these adaptations, as they represent near optimal osteogenic stimuli [58]. Further evidence of the favourable musculoskeletal potential of soccer movements comes from demonstration that the activity pattern and high intensity actions involved in soccer training provide marked increases in lower extremity [49,54] as well as upper body LBM [53], and that the whole-body muscle hypertrophic effects of soccer are greater than for continuous running and interval running, and as effective in increasing LBM as resistance exercise [59]. With regards to the cardio respiratory fitness effects of soccer, it has been shown that short-term soccer training was greater [60,61] or equal to [62] training volume-matched continuous running programs, and similar to the effects of high-intensity interval running [63]. Interestingly, soccer training was perceived as less exhausting than both continuous and intermittent running in young healthy men [40]. Recreational soccer therefore may constitute a highly motivating exercise-based rehabilitation intervention. Importantly, soccer training also provides peer-based psychosocial support and added individual social capital [64], which is likely to contribute to long-term adherence to training. Of note, the above-mentioned studies investigated men and women aged 18–55 years. Less information is available about the effects of soccer training for elderly (>65 years) subjects but recent studies have shown that heart rate is also high for elderly soccer players during small-sided games [56] and cross-sectional studies have provided evidence of elderly soccer players’ impressive cardiovascular and musculoskeletal health profiles, with rapid muscle force and postural balance scores equal to those of 30 years-old untrained men [65].

One particular aim of this study is to address whether an out-door intervention with little need for equipment can achieve effect sizes comparable to those of multimodal interventions requiring relatively expensive training facilities, i.e. resistance exercise machines and stationary bicycles. With a low cost to benefit ratio, potential positive results from the study may be disseminated to a broader population of men with PCa, in co-operation with existing community-based soccer clubs. This could potentially make an important contribution to the cancer care pathway for PCa patients and make a significant, positive impact on PCa survivorship both short- and long term.

Finally, a goal of the current research project is to build a bridge between the clinical environment and the existing expertise within exercise- and sports psychology and physiology in order to meet the legitimate demands from male cancer survivors for patient-centered and action-orientated interventions aimed at improved health [66]. Collaboration between health care specialties, i.e., oncology, urology, cardiology, psychology, physiotherapy and exercise physiology in the current study is crucial for its success and the results are likely to benefit the care and rehabilitation of PCa patients with possible favorable effects on long-term clinical outcomes.

## Abbreviations

ADT: Androgen deprivation therapy; BMC: Bone mineral content; BMD: Bone mineral density; DXA: Dual-energy X-ray absorptiometry; IVST: Interventricular septal wall thickness; LBM: Lean body mass; LV: Left ventricular; LVEDD: Left ventricular end-diastolic diameter; LVID: Left ventricular internal dimension; OGTT: Oral glucose tolerance test; PAT: Peripheral arterial tonometry; PCa: Prostate Cancer; PWT: Posterior wall thickness; QoL: Quality of life; RER: Respiratory exchange ratio; RHI: Reactive hyperemic index; RM: Repetition maximum; TDI: Tissue doppler Imaging; VO2max: Maximal oxygen uptake.

## Competing Interests

The authors declare that they have no competing interests.

## Authors’ contributions

JM and JFC developed the study concept and initiated the project together with MR and PK. KB, JFS, LJA, PRH, TH, LLA, EWH, KBC and JU assisted in further development of the protocol. JU drafted the manuscript. KB will provide access to patients. All authors contributed to and approved the final manuscript.

## Pre-publication history

The pre-publication history for this paper can be accessed here:

http://www.biomedcentral.com/1471-2407/13/595/prepub
